# The relationship between salivary amylase and the physical and psychological changes elicited by continuation of autogenic training in patients with functional somatic syndrome

**DOI:** 10.1186/s13030-017-0103-y

**Published:** 2017-06-28

**Authors:** Tadashi Kiba, Tetsuya Abe, Kenji Kanbara, Fumie Kato, Sadanobu Kawashima, Yukie Saka, Kazumi Yamamoto, Yasuyuki Mizuno, Junji Nishiyama, Mikihiko Fukunaga

**Affiliations:** 1 Department of Psychosomatic Medicin, Kansai Medical University, 2-5-1 Shinmachi, Hirakata-shi, Osaka Japan; 2 Department of Psychosomatic Medicine, Nishi Kyoto Hospital, 24 Goryo Mizoura-cho, Nishikyo-ku, Kyoto Japan

## Abstract

**Background:**

The aim of this study was to clarify the changes in biological measures during autogenic training (AT) sessions and the relationship between these biological measures and the changes in physical and psychological measures induced by continuation of AT in patients with functional somatic syndrome (FSS). We used the salivary amylase (SAMY) level, skin temperature of the finger (TEMP), subjective symptom scores, and psychological characteristics to assess these changes.

**Methods:**

We assessed 24 patients with FSS and 23 healthy controls before and after AT. We then conducted the same tests after the participants had practiced AT at home 1 and 2 months later.

**Results:**

The baseline SAMY levels in the first session were significantly higher in the FSS group than in the control group. However, this difference was not significant in the second and third sessions. The pattern of changes in TEMP induced by AT was not different between the FSS and control groups. Tension-anxiety and somatic symptoms in patients with FSS were improved by AT. In the FSS group, the baseline SAMY levels in the first session showed a significant negative correlation with the changes in the subjective symptom score and tension-anxiety score at baseline.

**Conclusions:**

The practice of AT, both during the first session and after 1 month of continuation, eased the dysregulation of the autonomic nervous system that is reflected in SAMY in patients with FSS. AT also contributed to decreases in the tension-anxiety and somatic symptoms in patients with FSS. We suggest that SAMY is related to both physical and psychological effects of AT in patients with FSS.

## Background

Functional somatic syndrome (FSS) encompasses a group of syndromes with a medically unknown origin [1, 2]. According to Barsky and Borus, FSS refers to “several related syndromes that are characterized more by symptoms, suffering, and disability than by disease-specific, demonstrable abnormalities of structure or function” [1]. Presently, there is no objective criterion with which to define FSS [3].

Although FSS includes a range of diseases such as irritable bowel syndrome (IBS), functional dyspepsia (FD), fibromyalgia syndrome (FMS), and chronic fatigue syndrome (CFS), there is considerable overlap among the symptoms characteristic for each disorder [4].

Patients with FSS often undergo repeated investigation and treatment in hospitals. Thus, FSS is associated with substantial costs to patients and the health system. FSS constitutes a large, clinically important, and costly health-care issue that urgently requires better understanding and improved management [2]. However, the pathological conditions of FSS remain poorly understood.

The conditions of FSS are strongly related to psychosocial factors [1]. The somatic symptoms of FSS are maintained, chronically prolonged, and deteriorated by mood disturbances, such as anxiety or depression [5]. Moreover, dysregulation of the autonomic nervous system (ANS) is also one of the important components of FSS [6]. In addition, dysregulation of the hypothalamic-pituitary-adrenal (HPA) axis is related to the pathological conditions of FSS [7].

Considering the above, it seems necessary to examine the pathological elements of FSS from multiple perspectives, including objectively, subjectively, and psychologically.

We previously examined dysregulation of the ANS in patients with FSS via psychophysiological evaluation. We found that patients with FSS had a hypo-reactive psychophysiological acute stress response compared with healthy controls [8]. In another previous study, we found that the physiological response in patients with FSS was lower than that in controls, but we identified two subgroups among individuals with FSS who differed in terms of autonomic lability (i.e., low- and high-lability subgroups) [9]. These studies suggest that evaluation of the autonomic activity is important when considering the pathological conditions of FSS.

Moreover, we evaluated the HPA axis in patients with FSS using salivary cortisol [10]. Our findings suggested that patients with FSS had a dysfunctional HPA axis, which might be a pathological cause of their persistent symptoms.

However, using cortisol as an endocrine marker has certain limitations. For instance, secretion of cortisol is usually delayed by 20 to 30 min after stress stimulation in a stress test [11]. In contrast, the measurement of salivary amylase (SAMY) can be used as an index of the sympathetic activity with the advantages that it is instantaneousness because the secretion is delayed by a few minutes after stress stimulation [12–14]. Some studies using SAMY have reported that its level increases in response to heightened psychophysiological stress and is positively correlated with states of anxiety [15–17].

We previously examined the pathological conditions of FSS using SAMY [18]. We found that the SAMY levels of patients with FSS were significantly higher than those of healthy controls, suggesting that SAMY is useful as an index of sympathetic activity in patients with FSS.

Autogenic training (AT) is one approach that can be used to adjust autonomic activity [19]. In addition, AT is effective for relieving physical symptoms associated with anxiety or depression [20]. AT decreases cardiac sympathetic activity and increases cardiac parasympathetic activity [21]. AT also increases the peripheral skin temperature [22], making the skin temperature of the finger (TEMP) a useful objective index for measuring changes induced by AT.

A previous study reported that AT is clinically effective for not only improving mood and cognitive performance but for treating tension headache, migraine, mild-to-moderate essential hypertension, and coronary heart disease [23].

Several studies have investigated the influence of AT on different types of FSS, such as IBS [24], FMS [25], and tension headache [26, 27]. In these studies, AT elicited changes in physical and psychological measurements such as the patients’ physical symptoms and quality of life. To the best of our knowledge, however, few studies have addressed the effect of AT on the full spectrum of FSS. We previously found that the baseline levels of SAMY prior to the first AT session were significantly higher in the FSS group than in the control group, and this difference between the two groups was not significant after AT [28]. Actually, it usually takes about 2 to 8 months to improve chronic symptoms by continuing AT [29]. However, we identified no studies that used SAMY to assess the accumulative effect of AT in patients with FSS.

Thus, the aim of this study was to clarify the changes in biological measures during AT and the relationship between these biological measures and the changes in physical and psychological measures induced by continuation of AT in patients with FSS. We evaluated these changes using the SAMY level, TEMP, subjective symptom score, and psychological characteristics.

## Methods

### Participants

Twenty-four patients (16 female, 8 male; age range, 22–78 years; mean ± standard deviation, 42.00 ± 15.36 years) comprised the participant group. All individuals were diagnosed with FSS according to the criteria described below.

For study eligibility, we selected outpatients and inpatients of the Department of Psychosomatic Medicine of Kansai Medical University Hirakata Hospital for whom physicians specializing in psychosomatic medicine (members of the Japanese Society of Psychosomatic Medicine) expected that AT would be effective based on the results of a psychophysiological assessment. All participants provided written informed consent.

A patient was diagnosed with FSS if he/she met the following four conditions, which were based on the diagnostic criteria from our previous study [28]: (1) chief complaints were somatic symptoms that could not be explained medically or by a psychiatric disorder, (2) a subjective symptom score ≥3 based on a visual analogue scale [30] and a ≥6-month duration of symptoms, (3) symptom-induce disabilities that affected social or daily activity (Global Assessment of Functioning Scale score [31] of ≤80), (4) and diagnosis of a disease with a review number of ≥2 in the study by Henningsen et al. [3]. We began with 34 potential participants. Based on above the criteria, five patients were excluded from the study (owing to the FSS criteria (4)). Two patients were excluded because they were being treated with β-adrenergic blockers, which are known to reduce the SAMY level [12]. One patient was excluded because she was being treated with a tricyclic antidepressant, which is known to increase the SAMY level [32]. Two patients dropped out during the AT procedure. Finally, the remaining 24 patients were entered into the analysis portion of the study.

The diagnoses of the 24 patients were as follows: IBS (*n* = 8), FD (*n* = 11), FMS (*n* = 4), CFS (*n* = 2), tension headache (*n* = 6), premenstrual syndrome (*n* = 2), chronic low back pain (*n* = 1), and globus syndrome (*n* = 1) (Table 1). The physicians made these diagnoses according to the criteria for each syndrome. FD and IBS were diagnosed using the ROME III criteria [33, 34], FMS using the American College of Rheumatology 1990 criteria [35], CFS using the International Chronic Fatigue Syndrome Study Group criteria [36], tension headache using the International Classification of Headache Disorders 2nd Edition [37], premenstrual syndrome using the ACOG practice bulletin criteria [38], chronic low back pain using the criteria described by Last and Hulbert [39], and globus syndrome using the criteria described by Koike et al. [40].

**Table 1:** Diagnoses of 24 patients

FD	11
IBS	8
Tension headache	6
FMS	4
CFS	2
Premenstrual syndrome	2
Chronic low back pain	1
Globus syndrome	1

*FD* functional dyspepsia, *IBS*: irritable bowel syndrome, *FMS* fibromyalgia syndrome, *CFS* chronic fatigue syndrome

Their mean period of attendance to the Department of Psychosomatic Medicine of Kansai Medical University Hirakata Hospital before the first AT session was 13.71 months (range, 1–48; standard deviation, 14.01), and it was not correlated with the baseline SAMY level in the first AT session (Pearson’s correlation coefficient (CC) = −0.236, *P* = 0.267).

We asked the participants to maintain the dosage of their existing medications prior to the examination, when possible, and they reported complying with this request. Six of the patients drank alcohol on a regular basis.

Twenty-three healthy participants (13 female, 10 male: age range, 16–65 years; mean ± standard deviation, 37.83 ± 10.87 years) participated as controls. They were recruited through a public announcement that asked people to participate in a study examining changes in autonomic activity induced by AT. Individuals who were regularly receiving medical care or had somatic symptoms were excluded. There was 1 underage participant, and her parent agreed with her participation based on approval by the ethics committee of Kansai Medical University. The controls were paid 3000 yen each for their participation. Ten of the healthy subjects drank alcohol regularly. We found no significant differences between the FSS group and healthy controls with respect to age (*t*-test; *P* = 0.075, d = 0.310), the male/female ratio (Fisher's exact test; *P* = 0.556), or the alcohol drinker/nondrinker ratio (Fisher's exact test; *P* = 0.227) (Table 2).

**Table 2:** Demographic data

	FSS	Control	*P* value (effect size)	
n	24	23		
Male/female	8/16	10/13	0.678	(0.104)
Age, mean (SD)	42.00 (15.36)	37.83 (10.87)	0.075	(0.310)
Alcohol drinkers/non-drinkers	6/18	10/13	0.304	(0.195)

*FSS* functional somatic syndrome

This study was approved by the ethics committee of Kansai Medical University.

### Autogenic training

The form of AT used in this study was based on the style developed by Schulz [29, 41, 42]. In this study, AT consisted of two standard exercises that took place after the participant stated the following formula: “I am at peace.” The first exercise encourages muscular relaxation via repetition of the formula, “My arms are heavy.” The next exercise encourages patients to feel warm via repetition of the following formula: “My arms are warm.” AT was performed in a sitting posture using a tape recording.

The participants completed three AT sessions at an interval of approximately 60 days. Each AT session was conducted individually in an examination room. During the periods between these three sessions, the participants were asked to complete 3 min of AT twice a day. The mean interval between the first and second sessions was 28.63 days (range, 21–43; standard deviation, 3.97) in the FSS group and 31.39 days (range, 20–47; standard deviation, 5.43) in the control group, which was not significantly different (*t*-test; *P* = 0.225, d = 0.360). The mean interval between the first and third sessions was 58.33 days (range, 45–70; standard deviation, 4.91) in the FSS group and 62.74 days (range, 50–77; standard deviation, 6.28) in the control group, again without a significant difference (*t*-test; *P* = 0.264, d = 0.330).

### Biological measurements

We measured the SAMY level using a hand-held SAMY monitor (Amylase monitor; Nipro Co., Ltd., Osaka, Japan). Amylase monitor enables a user to measure automatically the SAMY levels with high accuracy, using a dry-chemical system and 30-μl sample of saliva, within 1 min from collection to completion of the measurement. Saliva was collected by a test strip placed under the tongue for approximately 30 s, then immediately measured. The reliability and validity of Amylase monitor have been confirmed previously [11].

We measured the body temperature (TEMP) using a non-contact thermometer (Human Ful Thermometer; Mistal Co., Ltd., Sendai, Japan) because we were able to gain more detailed information about TEMP via infrared rays [43]. We measured the skin temperature of the tip of the middle finger of both hands and used the mean for analysis.

### Subjective symptom score measurements

We used a visual analogue scale to evaluate the subjective severity of symptoms in the FSS group. The scores ranged from 0 (absent) to 100 (most severe).

### Mood measurements

To evaluate the psychological characteristics of the participants, we administered the Japanese edition of the Profile of Mood States (POMS) [44] in the first and third sessions. The POMS is a 65-item scale that assesses 6 temporal affective mood dimensions. The subscale consists of tension-anxiety (T-A; range of *T*-score, 31–85), depression-dejection (D; range of *T*-score, 40–85), anger-hostility (A-H; range of *T*-score, 37–85), vigor (V; range of *T*-score, 27–80), fatigue (F; range of *T*-score, 35–85), and confusion (C; range of *T*-score, 32–85). The reliability and validity of the POMS have been confirmed [44].

### Procedure

This study was conducted at the Department of Psychosomatic Medicine of Kansai Medical University Hirakata Hospital in Osaka, Japan from July 2012 to March 2015.

**Fig. 1:**
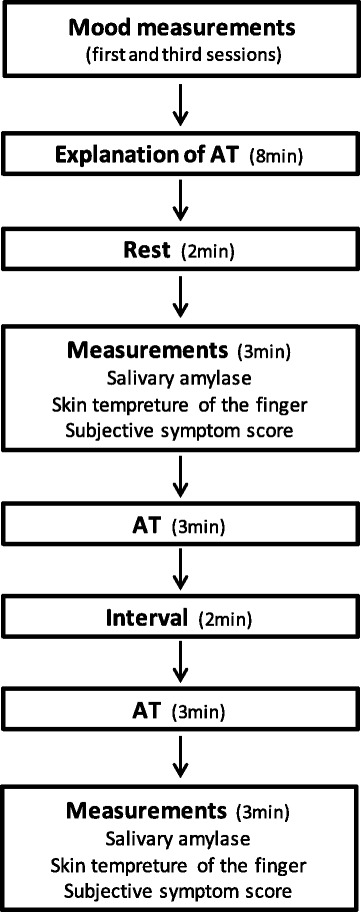
Mood measurements were collected at home on the day of the first and third sessions. Before starting the experiment, the participants received an explanation of AT. They were first instructed to relax for 2 min, and then they were instructed to engage in AT for 3 min. After an interval of 2 min, they repeated the AT. The SAMY level, TEMP, and subjective symptom score were collected both before and after AT

The session protocol is shown in Fig. 1. Mood measurements were collected at home on the day of the first and third sessions. Biochemical, psychophysiological, and subjective measurements were collected in a hospital examination room. The room temperature was kept constant at 25 °C. Before starting the experiment, the participants received an explanation about the effects and method of AT both in writing and on a video. They were first instructed to relax and make themselves comfortable for 2 min, and then they were instructed to engage in AT for 3 min. After an interval of 2 min, they repeated AT. We collected the SAMY level, TEMP, and subjective symptom score both before and after AT. Considering the circadian variations in the SAMY level [45], the examination was scheduled to take place between 15:30 and 16:30. Participants were required to avoid eating or drinking for 2 h before the examination to minimize the influence.

### Statistical methods

To compare changes in the SAMY level and TEMP, we conducted a three-way repeated-measures analysis of variance (ANOVA) in which one within-subjects factor was “point” (two levels: baseline and after AT), another within-subjects factor was “session” (three levels: the first, second, and third sessions), and the between-subjects factor was “group” (two levels: control and FSS groups).

To assess changes in the subjective symptom score, we conducted a two-way repeated-measures ANOVA in which one within-subjects factor was “point” (two levels: baseline and after AT) and another within-subjects factor was “session” (three levels: the first, second, and third sessions).

If the ANOVA results were significantly different, we used the Bonferroni correction to evaluate the significance of the individual differences.

We conducted an unpaired *t*-test in the first session to compare the psychological test results between patients with FSS and healthy controls. Additionally, we conducted a paired *t*-test between the first and third sessions to assess changes in the psychological test results in patients with FSS.

CC was used to analyze the relationship between the SAMY level/TEMP at baseline in the first session and the changes in the subjective symptom score/psychological test results at baseline between the first and third sessions.

Statistical analyses were performed using PASW statistics 18.0 for Windows (SPSS Inc., Chicago, IL, USA). The α level was fixed at 0.05.

## Results

### Salivary amylase

**Fig. 2:**
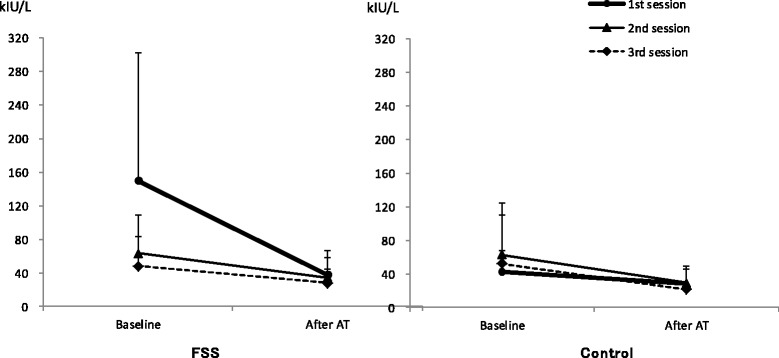
The baseline SAMY levels in the first session were significantly higher in the FSS group than in the control group. However, this difference was not significant in the second and third sessions

Figure 2 shows the changes in the SAMY levels in the three AT sessions in the FSS and control groups. The three-way ANOVA indicated that the point–session–group interaction was significant (F(2,90) = 7.921, *P* = 0.002, η_p_^2^ = 0.150).

The point–session interaction was not significant in the control group F(2,44) = 0.927, *P* = 0.403, η_p_^2^ = 0.040), but it was significant in the FSS group (F(2,46) = 7.519, *P* = 0.008, η_p_^2^ = 0.246).

In the FSS group, the point main effects were significant (F(1,23) = 28.167, *P* = 0.001, η_p_^2^ = 0.550). The simple main effects test revealed that the difference in the SAMY levels between baseline and after AT in the first session was significant (*P* = 0.001), but this difference was not significant in the second session (*P* = 0.316) or third session (*P* = 0.722). In addition, the SAMY level at baseline in the first session was significantly higher than that measured in the second session (*P* = 0.001) and third session (*P* = 0.001); after AT, however, there were no significant differences in the SAMY level between the first and second sessions (*P* = 1.000) or between the first and third sessions (*P* = 1.000). Therefore, our findings indicate that in the FSS group, the decrease in SAMY levels was significant during the first session but not during the second and third sessions.

In the control group, the SAMY level after AT was significantly lower than the baseline level (F(1,22) = 16.182, *P* = 0.001, η_p_^2^ = 0.424). However, we did not find a significant difference in the session main effects (F(2,44) = 1.251, *P* = 0.296, η_p_^2^ = 0.054).

### Skin temperature of the finger

**Fig. 3:**
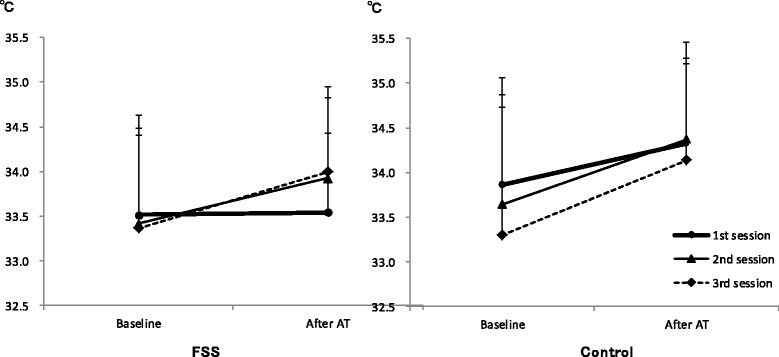
The pattern of changes in TEMP induced by AT was not different between the FSS and control groups

Figure 3 shows the changes in TEMP for the three AT sessions in the FSS and control groups. The three-way ANOVA indicated that the point–session–group interaction was not significant (F(2,90) = 0.490, *P* = 0.614, η_p_^2^ = 0.011). Thus, the pattern of changes in TEMP induced by AT was not significantly different between the FSS and control groups.

### Subjective symptom score

**Fig. 4:**
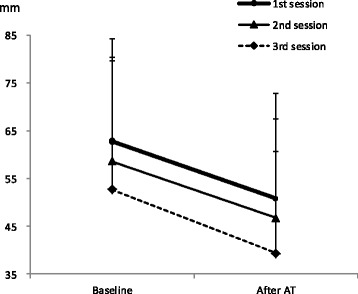
Somatic symptoms in patients with FSS were improved by AT

Figure 4 shows the changes in subjective symptom scores for the three AT sessions in the FSS group. The two-way ANOVA showed that the point main effects (F(2,23) = 24.085, *P* = 0.001, η_p_^2^ = 0.512) and session main effects (F(2,46) = 3.426, *P* = 0.041, η_p_^2^ = 0.130) had significant effects, but the point–session interaction (F(2,46) = 0.029, *P* = 0.935, η_p_^2^ = 0.001) was not significant. The subjective symptom scores in the FSS group decreased during each session and between the first and third sessions.

### Psychological test results

Table 3 shows the psychological characteristics recorded in the first session in the FSS and healthy control groups. As indicated by the *t*-test, patients with FSS exhibited significantly lower POMS–V scores, while all other psychological test scores in patients with FSS were significantly higher than those obtained by healthy controls. Table 4 shows the psychological characteristics recorded in the first and third sessions in the FSS group. As indicated by the *t*-test, patients with FSS exhibited a significant decrease in the POMS–T-A score between the first and third sessions.

**Table 3:** Psychological characteristics in the first session in the FSS and control groups

	Mean (SD) < range>							
	FSS (*n* = 24)			Control (*n* = 23)			*P* value (effect size; d)	
POMS								
T-A	62.33	(13.66)	<36–84>	46.22	(6.69)	<36–64>	0.000^**^	(1.490)
D	63.33	(12.76)	<42–82>	45.13	(6.28)	<40–65>	0.000^**^	(1.800)
A-H	54.92	(9.45)	<40–74>	48.52	(11.00)	<38–79>	0.038^*^	(0.630)
V	39.92	(7.58)	<29–61>	49.74	(10.69)	<35–80>	0.001^*^	(1.060)
F	60.46	(11.43)	<35–77>	50.61	(10.38)	<36–69>	0.003^*^	(0.900)
C	59.54	(13.37)	<38–83>	45.83	(8.06)	<34–66>	0.000^**^	(1.240)

^**^ *P* < 0.01

^*^ *P* < 0.05

*FSS* functional somatic syndrome, *POMS* Japanese edition of the Profile of Mood States, *T-A*, tension-anxiety, *D* depression-dejection, *A-H* anger-hostility, *V* vigor, *F* fatigue, *C* confusion

**Table 4:** Psychological characteristics in the first and third sessions in the FSS group

	Mean (SD) < range>							
	1st session			3rd session			P value (effect size; Δ)	
POMS								
T-A	62.33	(13.66)	<36–84>	54.79	(14.52)	<33–82>	0.035^*^	(-0.552)
D	63.33	(12.76)	<42–82>	60.54	(11.97)	<40–84>	0.267	(-0.219)
AH	54.92	(9.45)	<40–74>	51.33	(11.20)	<30–74>	0.215	(-0.380)
V	39.92	(7.58)	<29–61>	41.04	(11.31)	<28–78>	0.605	(0.148)
F	60.46	(11.43)	<35–77>	58.50	(11.74)	<35–77>	0.407	(-0.172)
C	59.54	(13.37)	<38–83>	57.67	(13.18)	<34–85>	0.546	(-0.140)

^*^ *P* < 0.05

*FSS* functional somatic syndrome, *POMS* Japanese edition of the Profile of Mood States, *T-A* tension-anxiety, *D* depression-dejection, *A-H* anger-hostility, *V* vigor, *F* fatigue, *C* confusion

### Correlation between salivary amylase/skin temperature and changes in the subjective symptom score/psychological test results

**Fig. 5:**
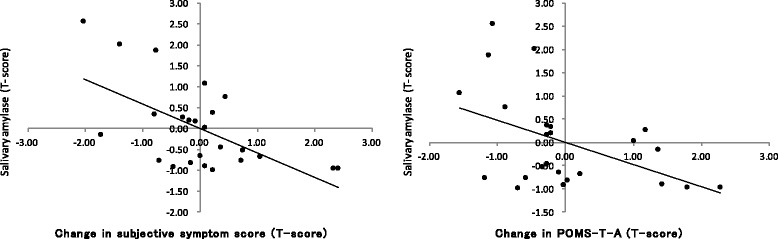
In the FSS group, the baseline SAMY levels in the first session showed a significant negative correlation with the changes in the subjective symptom score and tension-anxiety score at baseline

Table 5 and Fig. 5 show the relationship between SAMY/TEMP at baseline in the first session and the changes in the subjective symptom score/psychological test results at baseline between the first and third sessions in patients with FSS. In the FSS group, the SAMY level at baseline in the first session showed a significant negative correlation with the change in the subjective symptom score (CC = −0.599, *P* = 0.002) and the change in the POMS–T-A score (CC = −0.473, *P* = 0.020), but the TEMP was not correlated.

**Table 5:** Correlation between SAMY/TEMP at baseline in the first session and changes in the subjective symptom score/psychological test results at baseline in patients with FSS

	SAMY		TEMP	
	CC	*P* value	CC	*P* value
Subjective symptom score	-0.599	0.002^**^	0.111	0.607
POMS				
T-A	-0.473	0.020^*^	-0.060	0.779
D	-0.174	0.417	-0.298	0.158
AH	-0.201	0.346	-0.186	0.383
V	-0.110	0.610	-0.248	0.243
F	-0.403	0.051	-0.146	0.496
C	-0.101	0.639	-0.134	0.533

^**^ *P* < 0.01

^*^ *P* < 0.05

*SAMY* salivary amylase, *TEMP* skin temperature of the finger, *FSS* functional somatic syndrome, *CC* correlation coefficient, *POMS*: Japanese edition of the Profile of Mood States, *T-A* tension-anxiety, *D* depression-dejection, *A-H* anger-hostility, *V* vigor, *F* fatigue, *C* confusion

## Discussion

In the present study, we examined the changes in biological measures during AT sessions and the relationship between these biological measures and the changes in physical and psychological measures induced by continuation of AT in patients with FSS.

We found that the baseline SAMY level in the first AT session in the FSS group was significantly higher than that in the control group. This indicates that the sympathetic nervous system is strained in patients with FSS. This finding is in agreement with our previous study [28]. Moreover, the SAMY level under mental stress in patients with FSS was significantly higher than that in healthy controls in our previous study [18]. In addition, patients with FSS exhibit reduced cardiac vagal activity [6]. Considering the above, a highly strained sympathetic nervous system, as reflected by an elevated SAMY level at rest, may be associated with the pathological conditions of FSS.

In the FSS group, the SAMY level at baseline significantly decreased, not only during the first session but also between the first and second sessions. Although AT can reduce sympathetic hyperactivity [21, 28], it usually takes about 2 to 8 months to improve chronic symptoms by continuing AT [29]. We suggested that only one AT session as well as continuing it for 1 month can improve dysregulation of the ANS in patients with FSS.

We also observed a decrease in the SAMY level during each AT session in the control group. In our previous study, the SAMY level decreased during the first AT session in the control group, suggesting that AT is a helpful method of relaxation for healthy people [28]. In the present study, we confirmed this effect of relaxation not only in the first AT session but also after continuing AT for 4 or 8 weeks.

On the other hand, the pattern of changes in TEMP induced by AT in each session was not significantly different between the FSS and control groups. This result is in agreement with our previous findings in the first AT session [28]. With respect to stress, TEMP is an index of variability in autonomic activity [46], and it decreases if the sympathetic nervous system is strained [47]. In addition, TEMP is thought to be affected by both sympathetic and parasympathetic activity because increases in the skin temperature can be induced by stimulation of parasympathetic activity [48]. In the FSS group in the present study, the continuation of AT contributed to heightening of the sympathetic nervous system, which corresponded with an increased SAMY level, although it did not contribute to increased TEMP. Considering the above, the continuation of AT for 8 weeks might not be enough to increase the parasympathetic activity induced by AT. However, considering that the skin temperature is known to be affected by metabolism and diaphoresis [48], there is room for debate regarding the relationship between changes in TEMP induced by AT and the autonomic activity.

The practice of AT, during the first session as well as after its continuation for 1 month, improved somatic symptoms in patients with FSS. A meta-analysis by Stetter and Kupper [23] also showed that AT was effective in addressing somatic symptoms. Moreover, several studies have reported that AT is effective in treating patients with specific diseases within the spectrum of FSS, such as IBS [24] and tension headache [26, 27]. The results of the present study do not contradict these findings. Although, our study cannot be conclusive because of the small sample size, we argue that AT contributes to the improvement of somatic symptoms in patients with FSS as a whole.

Mood disturbances such as anxiety or depression are closely involved with the pathological conditions of FSS [9, 18, 28], and the continuation of AT contributed to the decrease in the POMS–T-A score in patients with FSS in the present study. The meta-analysis by Stetter and Kupper [23] showed that AT was effective for treating negative moods. In some previous studies, continuation of AT for 10 to 20 weeks improved not only the POMS–T-A score but also other POMS scores (D, A-H, C, and V) [49, 50]. However, the latter were not significantly improved over 8 weeks in the present study. Because these differences might have been because of the time periods of the studies, further examination is needed.

When we examined the relationship between physical and psychological changes induced by continuation of AT and the SAMY levels in patients with FSS, we found that the SAMY level at baseline in the first session had a significant negative correlation with the changes in the subjective symptom score and POMS–T-A score at 8 weeks. This suggests that patients with FSS showing a low baseline SAMY level in the first session may more readily achieve improvement in their physical symptoms and T-A induced by continuation of AT.

Considering the above, we suggest that the SAMY level can be used as an index of the change in autonomic activity caused by continuation of AT in patients with FSS. Although many studies have used psychophysiological measures such as the heart rate, skin conductance, and TEMP to measure changes in autonomic activity caused by AT (e.g., [19, 47, 49, 51–53]), we argue that the SAMY level was also useful as an index of the changes in sympathetic activity in our previous study [28]. In the present study, we argue that the SAMY level is related to both physical and psychological effects of AT in patients with FSS.

### Limitations of this study

Whether the observed change between baseline and after AT in patients with FSS was produced as a result of AT alone is debatable because we did not use a waiting-list control in patients with FSS. This problem also applies to the interpretation of the change between the first and third sessions.

We only measured SAMY and TEMP to evaluate changes in autonomic activity caused by AT in the present study. Further studies are needed in order to clarify the relationship of SAMY and other psychophysiological measures.

Moreover, our sample size was small and the diagnoses of patients were uneven. The pathological conditions of FSS will become clearer via examination of larger populations.

Additionally, we selected patients for whom physicians specializing in psychosomatic medicine expected that AT would be effective. Thus, our participant selection process was potentially biased. In addition, the patients were being treated with basic psychosomatic treatment, and we could not exclude the influence because the treatment includes multiple approaches such as medical treatment, exercise therapy, and intervention to regulate the patient’s life.

Furthermore, given that FSS is a diverse syndrome, some of the patients had multiple diagnoses. In addition, we could not exclude the influences of confounding factors other than age, sex, and alcohol consumption. In addition, the age was different between the two groups. While it has been reported that the SAMY level increases with age, the difference was not significant [54].

## Conclusions

The practice of AT, during the first session as well as after its continuation for 1 month, eased the ANS dysregulation that is reflected in the SAMY level in patients with FSS. AT contributed to a decrease in tension, anxiety, and somatic symptoms in patients with FSS. We suggest that SAMY is related to both the physical and psychological effects of AT in patients with FSS.

## Abbreviations

A-H: Anger-hostility
ANOVA: Analysis of variance
ANS: Autonomic nervous system
AT: Autogenic training
C: Confusion
CC: Correlation coefficient
CFS: Chronic fatigue syndrome
D: Depression-dejection
F: Fatigue
FD: Functional dyspepsia
FMS: Fibromyalgia syndrome
FSS: Functional somatic syndrome
HPA: Hypothalamic-pituitary-adrenal
IBS: Irritable bowel syndrome
POMS: Japanese edition of the profile of mood States
SAMY: Salivary amylase
T-A: Tension-anxiety
TEMP: Skin temperature of the finger
V: Vigor
